# Structural plasticity of Cid1 provides a basis for its distributive RNA terminal uridylyl transferase activity

**DOI:** 10.1093/nar/gkv122

**Published:** 2015-02-20

**Authors:** Luke A. Yates, Benjamin P. Durrant, Sophie Fleurdépine, Karl Harlos, Chris J. Norbury, Robert J.C. Gilbert

**Affiliations:** 1 Division of Structural Biology, Wellcome Trust Centre for Human Genetics, University of Oxford, Roosevelt Drive, Oxford OX3 7BN, UK; 2 Sir William Dunn School of Pathology, University of Oxford, South Parks Road, Oxford OX1 3RE, UK

## Abstract

Terminal uridylyl transferases (TUTs) are responsible for the post-transcriptional addition of uridyl residues to RNA 3′ ends, leading in some cases to altered stability. The *Schizosaccharomyces pombe* TUT Cid1 is a model enzyme that has been characterized structurally at moderate resolution and provides insights into the larger and more complex mammalian TUTs, ZCCHC6 and ZCCHC11. Here, we report a higher resolution (1.74 Å) crystal structure of Cid1 that provides detailed evidence for uracil selection via the dynamic flipping of a single histidine residue. We also describe a novel closed conformation of the enzyme that may represent an intermediate stage in a proposed product ejection mechanism. The structural insights gained, combined with normal mode analysis and biochemical studies, demonstrate that the plasticity of Cid1, particularly about a hinge region (N164–N165), is essential for catalytic activity, and provide an explanation for its distributive uridylyl transferase activity. We propose a model clarifying observed differences between the *in vitro* apparently processive activity and *in vivo* distributive monouridylylation activity of Cid1. We suggest that modulating the flexibility of such enzymes—for example by the binding of protein co-factors—may allow them alternatively to add single or multiple uridyl residues to the 3′ termini of RNA molecules.

## INTRODUCTION

The addition of a poly(A) tail to the 3′ end of a eukaryotic messenger RNA is an essential step for mRNA stability, export from the nucleus to the cytoplasm, and translational competence (1). But in addition to the nuclear poly(A) polymerases (PAPs) involved in pre-RNA maturation, a number of cytoplasmic or non-canonical, PAPs have been described in metazoans that regulate the length of the mRNA 3′ poly(A) tail and therefore transcript stability (2). Recently, a family of non-canonical poly(A) polymerase-related enzymes that instead add uridylyl ribonucleotides to 3′ ends have emerged as critical enzymes in RNA metabolism. These terminal uridylyl transferases (TUTs) have been shown to be involved in a number of regulatory pathways, for example U6 snRNA 3′ end processing (3), cell cycle-dependent histone mRNA decay (4), miRNA-directed RNA decay (5) and, more recently, miRNA maturation (6–8) and the regulation of mature miRNA silencing activity (9).

One such TUT family member, Cid1 from *Schizosaccharomyces pombe*, is a now well-characterized TUT, which was first demonstrated to be localized to the cytoplasm and to possess poly(A) polymerase activity *in vitro* (10), but more recently has been shown to possess TUT or poly(U) polymerase activity, *in vitro* and *in vivo* (10–12). The TUTactivity of Cid1 has been shown to be important in promoting mRNA degradation of polyadenylated and 3′ trimmed transcripts mediated by Lsm1–7 and the exonuclease Dis3l2 (12,13). Similarly, in mammals 3′ uridylated mRNAs and pre-let-7 miRNAs produced by TUT activity are also targeted for exonucleolytic degradation by Dis3l2 (14–16). The mammalian TUTs orthologous to Cid1, ZCCHC11 (TUT4) and ZCCHC6 (TUT7) have very recently been shown to uridylate mRNAs (17) as well as pre-let-7 miRNAs (6–8). Thus, mammals very likely possess a TUT-dependent mRNA degradation pathway similar to that found in fission yeast, although the subtle differences between the two systems require further investigation.

Even though terminal uridylylation of mRNA signals transcripts for degradation, there are subtle differences between length of the U-tail and the decay pathway employed. This difference is especially intriguing given that Cid1 is a robust poly(U) polymerase (PUP) *in vitro* adding long (∼50nt) tails (11), whereas the predominance of mono- and di-uridylylation of polyadenylated messages *in vivo* suggested that Cid1 may have distributive rather than processive polymerase activity (12). This is especially perplexing for Dis3l2-mediated decay as Dis3l2 itself recognizes and is stimulated by longer (∼13 nt) U-tails (16,18).

Cid1 has been extensively characterized both structurally and biochemically (19–22), revealing that a single histidine (H336) in the nucleotide recognition motif (NRM) is predominantly responsible for discriminating uracil over other bases via a mechanism that was proposed to involve two alternative conformations of the H336 side chain (21). The histidine ‘flipping’ mechanism was suggested to allow the detection of Watson–Crick edge of uracil through hydrogen bonding of H336 to two uracil-specific features of this pyrimidine base, that is the O4 carbonyl and cyclic amine. This provided a structural basis for the nucleotide selectivity of Cid1, which is able to select UTP over other nucleotides, even if the latter are in excess (11). Additionally, an asparagine residue (N165) has been proposed to recognize the substrate RNA 3′ nucleotide (22), whilst several lysine and arginine residues, clustered into three basic patches on the enzyme's surface, have been shown to participate in Cid1 RNA substrate binding (21). All of these identified interactions of Cid1 appear to be important for TUTase activity. Nevertheless, the biochemical and structural basis for the switching of Cid1 between PUP and TUT activities has so far not been extensively investigated. It has however been suggested that a ‘β-trapdoor’ feature which apparently helps to contain substrates within the enzyme's active site (21) and/or the length and sequence of target RNA substrate play a role (22).

In 2012, we reported the crystal structure of Cid1 refined to 3.0 and 3.2 Å, for UTP-bound and Apo forms, respectively. We have successfully improved the resolution of diffraction of our crystals (23), resulting in a higher resolution (1.74 Å) crystal structure of Cid1. This has allowed us to observe directly the H336-flipping mechanism, whereby the imidazole of H336 switches between two conformers, via an unanticipated double conformation of the D330 carboxylate side-chain. Furthermore, we can observe a closed conformation of Cid1 whereby the N-terminal domain (NTD) rotates with respect to the C-terminal domain (CTD) about a hinge (N164–N165), similar to a closed enzyme conformation observed in our previous study (21). We suggest that this represents an intermediate stage in a proposed mechanism by which a uridylated RNA product is ejected from the enzyme (21). Normal mode analysis of the Cid1 crystal structure suggests the enzyme to be generally flexible about this hinge. Using site-directed mutagenesis and activity assays we show that flexibility of the hinge is essential for multiple rounds of uridylation on a single RNA. We therefore propose that the extent of the domain motion of the enzyme, resulting in large conformational changes, is characteristic of the class of enzymes to which Cid1 belongs and helps explain the enzyme's distributive activity *in vivo*.

## MATERIALS AND METHODS

### Protein expression and purification

For all forms of Cid1 described in this manuscript protein expression and purification were essentially as described elsewhere (23). Cid1 mutants were generated as described in (21) using the primers

**Table:** 

Cid1-N164P F	TGTGATATTGGATTTCCCAATCGTCTAGCTATTC
Cid1-N164P R	GAATAGCTAGACGATTGGGAAATCCAATATCACA
Cid1-N164PN165P F	TGTGATATTGGATTTCCCCCTCGTCTAGCTATTCAT
Cid1-N164PN165P R	ATGAATAGCTAGACGAGGGGGAAATCCAATATCACA
Cid1-F88D F	GCTGAATTGGTAGCCGATGGAAGTTTGGAATC
Cid1-F88D R	GATTCCAAACTTCCATCGGCTACCAATTCAGC

### Data collection and structure determination

An RNA-binding mutant of tCid1 was purified analogously to the wild-type enzyme (21) and was crystallized in 18% (w/v) PEG 3350 or 16% (w/v) PEG8000, 100 mM sodium citrate tribasic dihydrate, pH 5.5, at room temperature using nanolitre sitting drop vapour diffusion. Crystals were cryo-protected by the addition of glycerol in a stepwise manner to a maximum concentration of 25% (v/v) and were mounted in LithoLoops™ (Molecular Dimensions Ltd) before flash-freezing in liquid nitrogen. Diffraction data were collected on beamline I02 at Diamond Light Source, Didcot, Oxfordshire, UK, using a Pilatus 6M detector. Several crystals were tested for diffraction revealing two distinct crystal forms, of space groups *P*1 and *P*2_1_ and with unit cell parameters of *a* = 58.96, *b* = 62.26, *c* = 65.5 Å, *α* = 76.3°, *β* = 81.1°, *γ* = 63.2° and *a* = 62.7, *b* = 103.7, *c* = 76.3 Å, *β* = 110.8°, respectively. The highest resolution diffracting crystals yielded data to 1.74 and 2.52 Å for the *P*1 and *P*2_1_ crystal forms, respectively. Data were processed using XDS and SCALA within the Xia2 program package during data collection at the beamline, resulting in two complete (>95% in the highest resolution shell) datasets with an overall *R*_merge_ = 2.8% for the P1 crystal form, and 3.2% for the *P*2_1_ crystal form (see Table 1 for details). Both crystal forms were found to possess two molecules per asymmetric unit. The crystal structures were determined by molecular replacement using Phaser (24), in the CCP4 suite (25), with Chain A of the Apo tCid1 structure (PDB entry 4e7x, (21), RMSD 1.0 Å) as a search model. The MR calculations gave a single solution after locating two molecules in the asymmetric unit in space groups *P*1 and *P*2_1_ for the respective datasets.

**Table 1.** Data collection and refinement statistics

**Data collection**		
Crystal	tCid1 (K133A/R137A/R277A/K282A mutant) crystal form I	tCid1 (K133A/R137A/R277A/K282A mutant) crystal form II
Beamline	DLS I02	DLS I02
Wavelength (Å)	0.9795	0.9795
Temperature (K)	100	100
Unit cell (Å, °)	*a* = 58.96, *b* = 62.26, *c* = 65.5, *α* = 76.3, *β* = 81.1, *γ* = 63.2	*a* = 62.7, *b* = 103.7, *c* = 76.3, *α* = *γ* = 90, *β* = 110.8
Space group	*P*1	P2_1_
Resolution (Å)	45.90–1.74 (1.77–1.74)	58.83–2.52 (2.60–2.52)
Observed reflections	279932	102562
Unique reflections	79749	30480
Data completeness (%)	96.3 (95.5)	98.4 (98.7)
Redundancy	3.5 (3.4)	3.4 (3.5)
*I*/*σI*	17.0 (2.1)	23.2 (2.1)
*R*_merge_	0.028 (0.493)	0.032 (0.531)
CC_1/2_	0.999 (0.783)	0.999 (0.719)
**Refinement**		
Resolution (Å)	45.90–1.74	58.83–2.52
Number of reflections	79739	30460
Number of atoms		
Protein	5186	5840
Glycerol	12	N/A
Water	373	31
*R*_work_ (%)	17.43	17.61
*R*_free_ (%)	20.28	21.83
CC*	1.00 (0.937)	1.00 (0.915)
CC_work_/CC_free_ (highest shell)	0.88/0.81	0.92/0.83
RMSD from ideal geometry		
Bond lengths (Å)	1.071	0.881
Bond angles (°)	0.009	0.004
Mean *B*-factor (Å^2^)		
Protein	43.76	73.41
Glycerol	58.81	N/A
Water	50.22	58.43
Residues in favoured regions of Ramachandran plot (%)	98.42	98.37
Residues in allowed regions of Ramachandran plot (%)	1.68	1.63
MolProbity validation		
MolProbity score	0.98 (100th Percentile)	1.61 (99th percentile)
MolProbity clashscore	2.12 (99th Percentile)	4.96 (99th percentile)

### Structure refinement

After MR the refinement of the model in the medium resolution dataset (2.52 Å, *P*2_1_) was performed using REFMAC (26) and Phenix (27) using TLS and non-crystallographic symmetry (NCS) restraints. The resulting model was manually adjusted, with some regions rebuilt, in Coot (28). Refinement in Phenix yielded good final R-factors, (*R*_work_ = 19.03% and *R*_free_ = 23.01%). For the high-resolution dataset a restrained refinement of the model was performed using REFMAC (26) after MR with Phaser. Observing the resulting high-quality electron density map in Coot (28) it was clear that the NTD of one of the chains (Chain B) was not correctly placed—we presumed due to domain motion. Therefore, we performed density modification with Parrot (29) and automatic model building using Buccaneer (30) within the CCP4 suite in order to re-build the translocated domain. The structure was subsequently refined with Phenix using NCS with translation libration screw (TLS) restraints. The excellent quality electron density suggested several side chains possessed split conformations, and these were adjusted manually in Coot (28) and the occupancy set to 0.5 before refinement of their occupancies with Phenix. We also used the Feature Enhanced Map (FEM) routine in Phenix to further suggest side-chain conformations. During the final rounds of structure refinement using Phenix, NCS restraints were relaxed to allow the differences between the two chains to become apparent. The final structure possessed good *R*-factors (*R*_work_ = 17.43% and *R*_free_ = 20.28%) as well as excellent model geometry (see Table 1). We also assessed the validity of our structures to the diffraction data using the modified correlation coefficient of half datasets, CC* (31). CC*, CC_work_ and CC_free_ were calculated for the final structures in Phenix (see Table 1) and suggest that the structures were not overfitted. Both structures possessed zero Ramachandran outliers with 100% of the residues within allowed regions, as assessed by MolProbity (32) and have been deposited in the RCSB PDB with accession codes 4ud4 (*P*_1_, 1.74 Å structure) and 4ud5 (*P*2_1_, 2.52 Å structure). Structural figures were prepared using PyMol (Delano Scientific).

### Normal mode analysis (NMA)

Normal mode analysis of Cid1 was performed using the online tool WEBnm@ (33). The vibrational movements of Cid1 produced by WEBnm@ were visualized as vector fields using VMD (34). The lowest six frequency modes (modes 1–6) were ignored as they represent rigid-body translation or rotation and do not show conformational dynamics. The subsequent lowest frequency normal modes (modes 7–12) can differentiate between rigid structural domains and flexible regions of the protein and therefore infer movements (i.e. bending and torsion) that are natural to the structure and are non-trivial. NMA analysis was performed separately using the Apo crystal structures presented here.

### *In vitro* RNA nucleotidyl transferase assays

Polymerization reactions were set up as described in (21). Samples were separated on 12% acrylamide/8M urea gels.

## RESULTS AND DISCUSSION

We used an RNA-binding-defective mutant of Cid1 (K133A/R137A/R277A/K282A) to improve the resolution of our crystal diffraction for higher resolution studies. The structure determined to 1.74 Å contains two molecules in the asymmetric unit with one chain exhibiting a typical Apo conformation and the other having a significantly different conformation. Here, we report principally the detailed insights gained from the higher resolution crystal structure of Cid1 from each molecule.

### Higher resolution structural insights into Cid1

**Figure 1.**
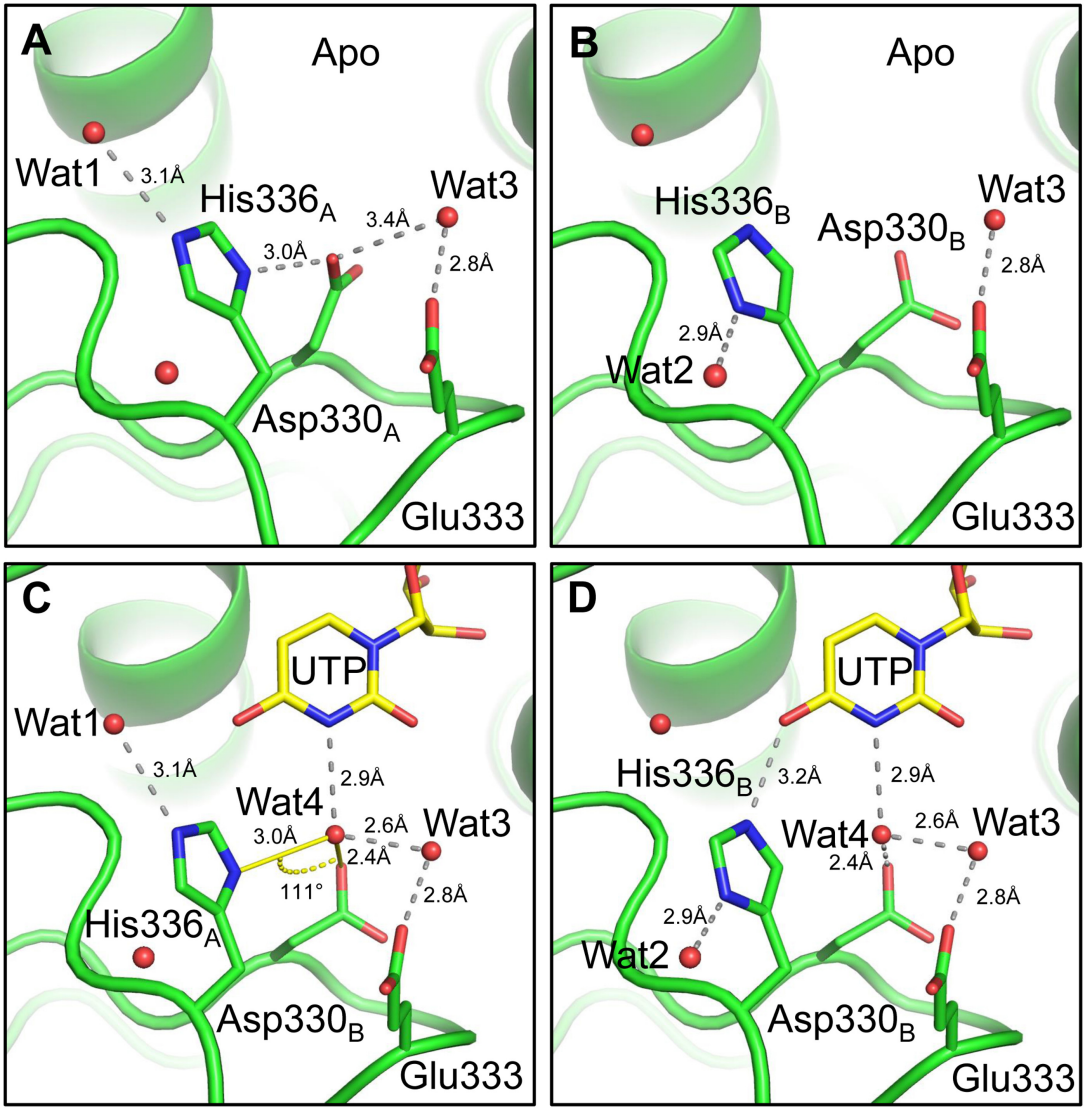
Detailed view of the NRM and the two conformations of H336 and D330 and their interaction network. In our higher resolution crystal structure we can observe that (**A**) the Nδ1 amine of the imidazole H336 conformer A (H336_A_) contacts Oδ1 of D330 and the Nϵ2 nitrogen contacts Wat1 (*B*-factor 65 Å^2^), whereas (**B**) the Nδ1 amine of H336 conformer B (H336_B_) contacts Wat2 (B-factor 62Å^2^). Both water *B*-factors are consistent with other waters in the surrounding area. We identify a proposed mechanism by which H336-flipping allows UTP selectivity, whereby (**C**) Wat4 is contacted by H336_A_ and D330_B_ with the angle between the H-bond vectors measuring 111° to allow the recognition of the uracil-specific cyclic amine through a donor:acceptor network. (**D**) H336_B_ contacts the uracil carbonyl and Wat4 forms a network with D330 and Wat3, whilst still maintaining an interaction with uracil. UTP and Wat4 were modelled from pdb deposition 4ep7 (20) after structure superposition of that and our model. Figures and measurements were made using PyMol (DeLano Scientific).

The higher resolution structure of Cid1 was originally sought for substrate interaction studies, however even the unliganded structure reported here has provided unexpected new detail about the interrelationship of structure and function in cytoplasmic uridylyltransferases. Several structures of Cid1 have been solved in a UTP-bound state (19–21) which demonstrate that residue H336 is critical for the uracil selectivity, with mutation to either Ala or Asn resulting in the conversion of TUTase activity to poly(A) polymerase activity (19,21). H336 is in close proximity to the Watson–Crick edge of the uracil moiety, with the Nϵ2 amine of the imidazole side chain contacting the carbonyl oxygen (O4) (19–21). However, structure validation of our UTP-bound structure (pdb 4e80) using a reduced model and MolProbity (32) suggested that half of the H336 side chains in the asymmetric unit were most likely in an alternative (flipped) conformation (21). This led us to propose a flipping mechanism that, along with a water molecule structurally conserved in related enzymes (35–37), might enable recognition of both the cyclic amine (N3) and the carbonyl (O4) of uracil (21). In one of the molecules in the asymmetric unit of our high resolution structure (chain A), presented here, we observe that indeed H336 adopts two conformations *via* a 180° rotation about the *χ*2 bond (Figure 1A and B). Unexpectedly, this H336 flipping occurs as a result of a double conformation of the D330 side chain, which was clearly visible in the unbiased electron density (Figure 1A and B, Supplementary Figure S1). Thus, in one orientation (conformer A), the imidazole is positioned such that the Nδ1 amine is 3.01 Å from the Oδ1 of D330, whilst the Nϵ2 amine contacts a water molecule some 3.04 Å away (Figure 1A). Whereas in a second conformation (conformer B) the H336 imidazolium group is positioned so that the Nϵ2 amine is able to recognize the carbonyl of UTP in the active site, whilst the other amine (Nδ1) is in close proximity (3.03 Å) and hydrogen bonds with a water molecule (Figure 1B). We initially refined the occupancies of the D330 double conformation, which suggested that each conformer (also denoted as A and B) was occupied equally (occupancy 0.5 each). The presence of two water molecules and the clear hydrogen bonding network that needs to be satisfied between D330 and H336, suggested that a split H336 side-chain be modelled and its occupancy refined. Satisfyingly, the occupancy of the H336 conformers was also equal (occupancy 0.5). Therefore, the H336 flipping previously reported (21), which was inferred by analysis of the refined structure, has now been observed directly and can be explained by the hydrogen bonding network with D330 and several water molecules.

In our earlier study, we proposed that the H336 flipping could ‘decode’ uracil by contacting the Watson–Crick edge of the base, that is the carbonyl oxygen (O4) and cyclic amine (N3) (Figure 1C and D). However, this mechanism relied on a water molecule that would, through a specific hydrogen bonding donor:acceptor network, detect the uracil N3 amine—analogous to a mechanism found in the trypanosomal enzyme RET2 (35). Using structural superposition between our high-resolution structure and a UTP-bound structure where waters are observed (pdb: 4ep7, (20)), we found that our H336_conformerA_ Nδ1 could contact a water molecule (Wat4; Figure 1C) modelled from the UTP-bound structure that is positioned 3.0 Å away and sits under the uracil N3 amine. The angle between our H336_conformerA_ Nδ1 to Wat1 contact vector and the Wat1 to D330 Oδ1 contact vector is ∼111°. This measurement is very close to the 109° bond angle of water and suggests that, in this conformation, the two hydrogens of Wat4 are donated to the H336 Nδ1 nitrogen and D330 carboxyl oxygen via hydrogen bonding. This donor:acceptor network results in the oxygen of Wat1 being only able to accept hydrogen bond donors, which in this instance is the hydrogen atom of the N3 amine of uracil. The lack of ideal hydrogen bond angles is likely due to the participation of Wat3 in the network. The observed rotation of the H336 side chain provides a structural basis for its significant role in UTP selectivity and supports previous biochemical data (19,21).

### Domain motion of Cid1 closes the active site

**Figure 2.**
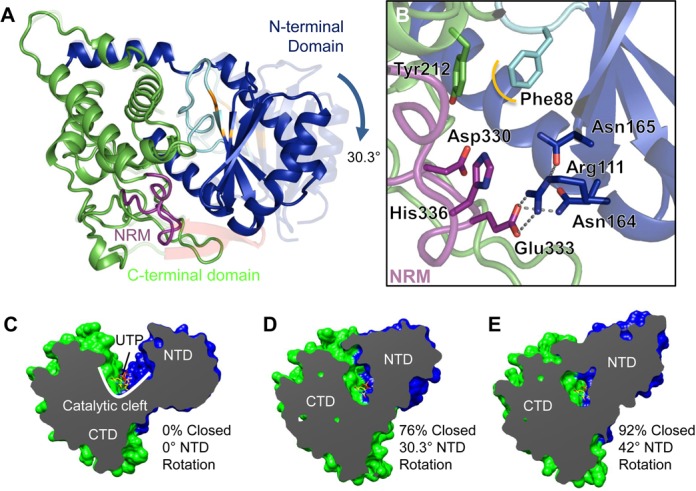
Domain rotation closes the active site. (**A**) The structure of an ‘intermediate’ closed enzyme. Comparison between Apo Cid1 ((21); pdb 4e7x) and the closed conformer we observed in our high resolution crystal structure, whereby the NTD rotates by approximately 30° with respect to the CTD. The N-terminal domain is rendered blue, the C-terminal domain is rendered green, the catalytic aspartates are coloured orange and the NRM is coloured purple. (**B**) A detailed view of the closed enzyme reveals that several key residues involved in interactions with the substrates are brought into close proximity and form an interaction network with the enzyme itself. (**C–E**) Dyndom analysis of the domain rotation and closure of Cid1. (**C**) A cutaway view of the enzyme (β trap door toward the reader) showing the deep catalytic cleft and bound UTP (pdb 4e80) for comparative purposes. (**D**) An ‘intermediate’ closed Cid1 (this report) whereby the 30.3° N-terminal domain rotation closes the active site by 76%. (**E**) A previously-observed closed conformer (pdb 4e8f) whereby the NTD rotates by 42° and closes the active site by 92%. UTP is modelled in all structures to illustrate that the domain closure could exclude substrate/product from the active site.

In a previous crystal structure, we observed a rotation of the NTD with respect to the CTD along with a remodeling of the catalytic region (21). The conformational change was substantial, with the NTD rotating 42° about a pivot point (residue range 163–166) to close the catalytic cleft. Of the two molecules in the asymmetric unit of the high resolution crystal structure described in this paper, it was clear from the excellent quality unbiased electron density that the NTD of chain B had adopted an unusual conformation. This conformation is related to one seen in our previous report (21) but the mutant enzyme used for this study is trapped in an intermediate stage compared to the previous closed form (Figure 2A and B). Analysis of this new closed form by Dyndom (38) revealed the NTD rotated by 30.3° compared to the CTD, thus closing the cleft by 76% (Figure 2A and D), whereas in the previous structure (pdb: 4e8f) the domain rotation closes the cleft by 92% (Figure 2E). Dyndom analysis indicates a pivot point for the domain motion of this ‘intermediate’ structure as residues 163–172, supporting the idea that there is an inherent flexibility within the enzyme between the two domains and that there is a single hinge point about which it occurs. We analysed the volume of the catalytic cleft of Cid1 and its intermediate closed conformer using 3V (39), and found that in a native open conformation the catalytic cleft volume accessible by solvent is 3339Å^3^ whereas the NTD rotation reduces the volume of the catalytic cleft by approximately a factor of 10 (336 Å^3^). The molecular details of catalytic cleft closure are discussed in detail below. We could not meaningfully perform volume analysis of the catalytic cleft in the previously described closed form because its model is incomplete, since elements of the structure are disordered.

### The structural plasticity of Cid1 and TUT activity

**Figure 3.**
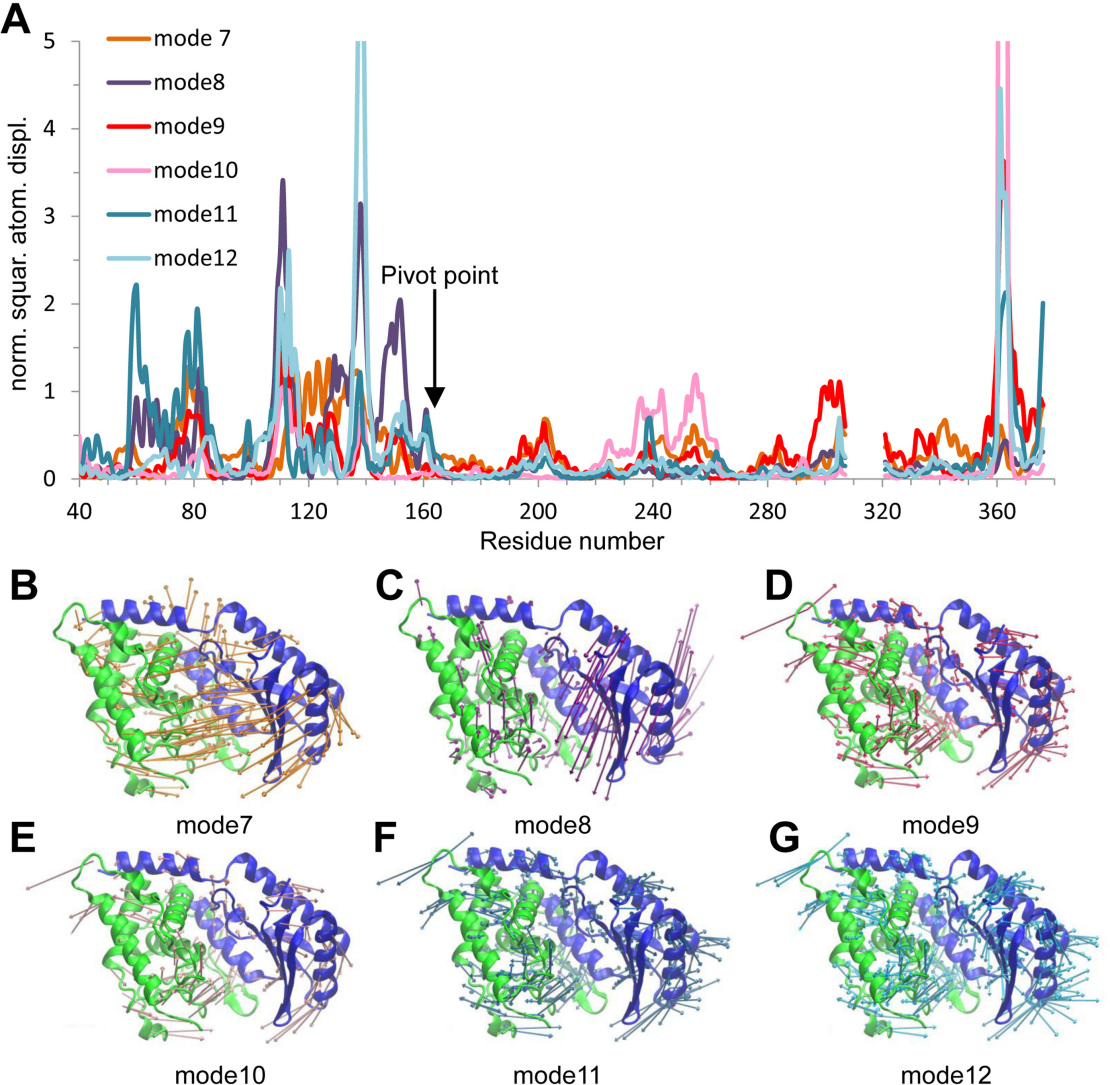
Normal Mode Analysis of Cid1. (**A**) Atom displacement (RMSD) of residues for the six lowest non-trivial vibrational modes (modes 7–12). Large motions (those off the scale) are correlated with termini and flexible loop regions of Cid1. Overall the majority of the N-terminal domain (residues 40 - 165) is more flexible than the C-terminal portion. (**B–G**) Vector field representations show the direction and magnitude (larger vectors show greater motion) of movement of each residue in the structure. (**B**) Normal mode 7 demonstrates a bending motion with correlated motion between the two domains towards the active site. (**C**) Normal mode 8 demonstrates torsional motion of the N-terminal domain, with respect to the CTD. (**D**–**G**) The remaining four of the six non-trivial modes demonstrate a flexing of the NTD generally moving inward toward the active site. Images were rendered using VMD (34) and coloured as in Figure 2. RMSD plots were generated with WEBnm@ (33). Vectors are colour coded for each mode and correspond to their respective RMSD plots.

Cid1 is capable of small ‘breathing’ motions and employs an induced fit mechanism when binding and recognizing UTP (21). Furthermore, Cid1 is also capable of large conformational changes that result in the closure of the enzyme (see above). We hypothesized that the flexibility of Cid1, and therefore its structural plasticity, could, in part, explain its predominant mono- and di-uridylyltransferase activity observed *in vivo* (12). To this end, we performed normal mode analysis of the Cid1 crystal structure using WEBnm@ (33) to assess its motional properties. The first non-trivial mode (normal mode 7) shows that the NTD flexes about the αE–β5 loop (residues 164–166) compared to the CTD. Indeed, plotting the atomic displacement of each mode from NMA shows that overall the NTD is more flexible (Figure 3A). Furthermore, there is an appreciable difference in the magnitude of atomic displacement between the N-terminal portion (residues 40–165) and the C-terminal half (Figure 3B), suggesting that the rotation involved is centered around residues 160–165, again supporting the notion that Cid1 has a hinge region at this point. Normal mode 8 demonstrates torsional motion suggesting that the NTD can rotate across its interface with the CTD as well as towards it (Figure 3C).

We next considered the flexibility of other known TUTs, most notably those from trypanosomes, performing normal mode analysis on TUT4, RET2 and MEAT1 to assess the degree of flexibility of these enzymes and whether structural plasticity is an evolutionarily conserved feature of TUTs in general (Supplementary Figure S2). Each TUT investigated demonstrated a level of plasticity similar to that of Cid1, with both bending and torsional motions identified. A comparison of the atom displacement plots suggests that Cid1 is similarly flexible to *Tb*TUT4 and *Tb*MEAT1 with the NTD exhibiting, in general, more flexibility than the C-terminal portion (Supplementary Figure S2A, B and C). Surprisingly, the most flexible domain of *Tb*RET2 is the so-called middle domain (residues 153–262) that is inserted within the NTD (Supplementary Figure S2D).

### Characterising the interdomain hinge of Cid1

**Figure 4.**
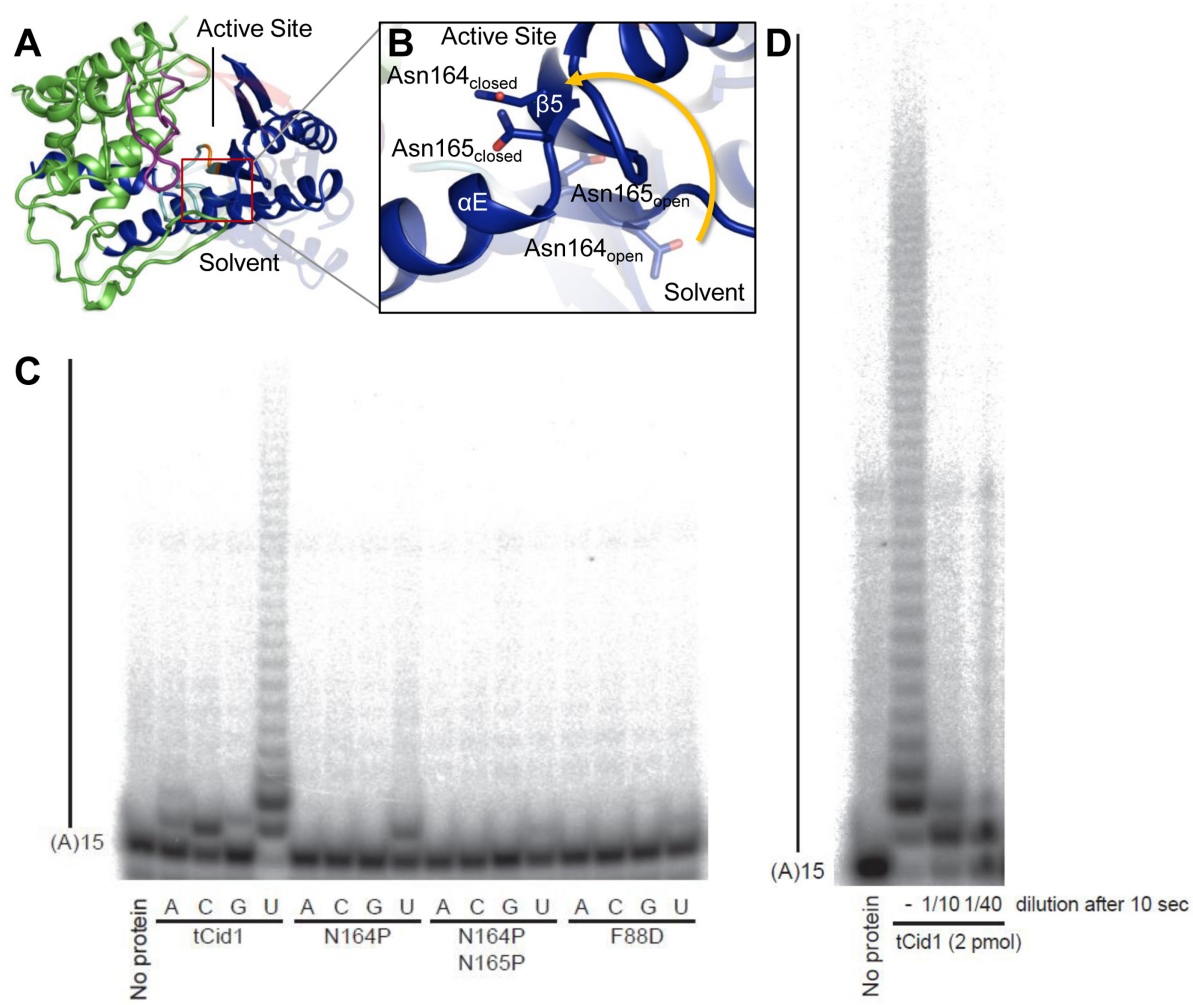
Domain movement about a hinge region in Cid1 is critical for activity. (**A**) Overall structure of Cid1 (coloured as in Figure 1) showing the hinge region (boxed) with (**B**) a detailed view of the loop between helix αE and the strand β5 illustrating that upon enzyme closure both N164 and N165 move significantly. N164 exhibits the greatest motion and also flips from a ‘*trans*’-like side chain configuration, with respect to N165, to a ‘*cis*’ like configuration. (**C**) *In vitro* activity assay using tCid1 and its mutants and a 5′-end–labelled (A)_15_ RNA substrate. Products were resolved by denaturing PAGE. Mutation of the residues at the hinge region (N164/N165) to the less flexible proline results in a mono-uridylylation activity for N164P and diminished TUTase activity for N164P/N165P. In addition, F88D also demonstrates diminished TUTase activity. (**D**) Cid1 is a distributive TUTase. A reaction mix was set up for three samples with a concentration of 200 nM for tCid1 in binding buffer (20 mM Tris pH 7.8, 150 mM KCl, 2 mM MgCl_2_, 5% glycerol) supplemented with 0.5 mM UTP. This reaction was then split in three tubes. One sample was left untreated (–). One sample was diluted ten times in binding buffer supplemented with 0.5 mM UTP (final concentration for tCid1 20 nM, 1/10). The last reaction was diluted 40 times in binding buffer supplemented with 0.5 mM UTP (final concentration for tCid1 5 nM, 1/40). The reactions were then incubated for 20 min at room temperature.

The structural comparison between the Apo or UTP-bound structures of Cid1 and its closed forms and the normal mode analysis described above strongly indicate that residues 164–166 are the central pivot point of the domain rotation. Detailed comparison of the open and closed forms of the enzyme showed that N164 undergoes a large conformational change when the enzyme closes (Figure 4). Both N164 and N165 move significantly, however N164 appears to flip from a ‘*trans*’-like side chain configuration with respect to N165, whereby the N165 side chain faces the active site and the N164 side chain faces the solvent in the opposite direction, to a ‘*cis*’-like configuration, whereby both side chains of N164 and N165 enter the active site (Figure 4 and Supplementary Movie 1). Direct comparison of the Cid1 structure with those of TUTs from trypanosomes (Supplementary Figure S4) alongside the normal modes analysis described above suggests that a similar conformational change occurs in the reactive cycle of those enzymes, in particular *Tb*TUT4. In those cases the amino acids at the equivalent position to N164 are tyrosine in *Tb*TUT4 and phenylalanine in *Tb*RET2 (Supplementary Figure S4), which may have additional steric effects on domain closure, such as affecting its rate. Sequence alignment identifies a similar hinge in the mammalian TUTs ZCCHC6 and ZCCHC11, where the residue equivalent to Cid1 N164 is again a tyrosine, like in *Tb*TUT4; the presence of an asparagine in Cid1 itself rather than a bulky hydrophobic residue likely confers specific features on its activity. Interestingly, the human non-canonical poly(A) polymerase PAPD1 (TUTase1) possesses an asparagine doublet analogous to that at the hinge region of Cid1. On the other hand, the *Trypanosoma brucei* minor-editosome associated terminal uridylyltransferase 1 (*Tb*MEAT1) possesses a glycine within the hinge region, which could make the enzyme more flexible, but its possession of a bridge domain bracing the other two domains may rigidify the enzyme all the same (40). Normal mode analysis also indicates that *Tb*MEAT1 is more rigid than the other TUTs.

To investigate the flexibility of Cid1, particularly at the hinge region, we mutated N164 to a proline residue in order to restrict the motion of the enzyme. We observed in a TUTase activity assay with an (A)_15_ RNA substrate that the N164P mutant predominantly performed mono-uridylylation, in contrast to the wild-type enzyme, which demonstrated robust PUP activity (Figure 4). A double proline substitution of N164 and N165 resulted in defective TUTase activity. This suggests that the degree of flexibility is critical for product release but also that the N164P mutant can only undergo a single catalytic cycle. We considered this further by mutating F88 to alanine, as the crystallographic evidence suggests this residue contributes to product dissociation. We found that the F88D mutant possessed defective TUTase activity suggesting that it is primarily involved in UTP binding first and foremost with product release an additional function of this residue. Our data support and build on a suggested swivel motion of Cid1 occurring throughout the catalytic cycle (14,15) and complement data showing that a Cid1 N165A mutant is unable to add more than one nucleotide in an activity assay (22). These data, taken together with our crystallographic and structural analysis, suggest that Cid1 is predominantly a distributive mono-uridylylation enzyme requiring uridylylated RNA release before engaging in another catalytic cycle. We further assessed the distributive activity of Cid1 *in vitro* by diluting the enzyme in a TUTase activity assay (Figure 4D). The fact that Cid1 was unable to add more than two nucleotides to the (A)_15_ RNA after dilution and incubation for 20 min again suggests the enzyme has a distributive activity, adding one or two uridines to its substrate before releasing it. The long U tails observed *in vitro* would be the result of multiple associations of the enzyme to a substrate due to the high concentration of substrate and enzyme in the reaction.

### The plasticity of the β-trapdoor

A β-trapdoor structure (residues 310–322) was previously observed bridging the N- and C-terminal domains of Cid1 in a UTP-bound crystal structure and was suggested to play a role in UTP selectivity and enzyme function via substrate containment as part of an induced-fit mechanism (21). Deployment of the trap door correlated with H336 side chain flipping but its absence from the Apo crystal form and other UTP-bound forms suggested that its stabilization depends on direct contact with the CTD (21). On the other hand, biochemical studies of Cid1 without its β-trapdoor demonstrated a more distributive (less processive) poly(A) polymerase activity with altered NTP binding but unhindered RNA binding capacity (22). In addition to the novel structure (crystal form I, space group *P*1) described in detail above we were able to crystallize the RNA-binding mutant of Cid1 in yet another crystal form (crystal form II, space group *P*2_1_; see Table 1, Supplementary Figure S3), which is also an Apo state but this time with an observable β-trapdoor. However in this case the β-trapdoor does not bridge the two domains but instead is stabilized by contacting a crystallographically-related molecule. Normal mode analysis of the model, complete with its β-trap door, demonstrates that this feature is comparatively more flexible than the majority of the CTD (Supplementary Figure S2A). This strongly suggests that the ordering of the trap door which we have observed previously (21) is fundamentally a property of interaction with the NTD and not of NTP binding itself, because it can be mimicked by a crystal contact. We suggest that this enables the trap door to act as a ‘strut’ preventing the collapse of the catalytic cleft when enzyme substrates are bound but before uridylylation has occurred. The β-trap door is unique so far to Cid1 among described cytoplasmic uridylyltransferases, being absent from the homologous ZCCHC6 and ZCCHC11 enzymes.

### A model for the catalytic cycle of Cid1

**Figure 5.**
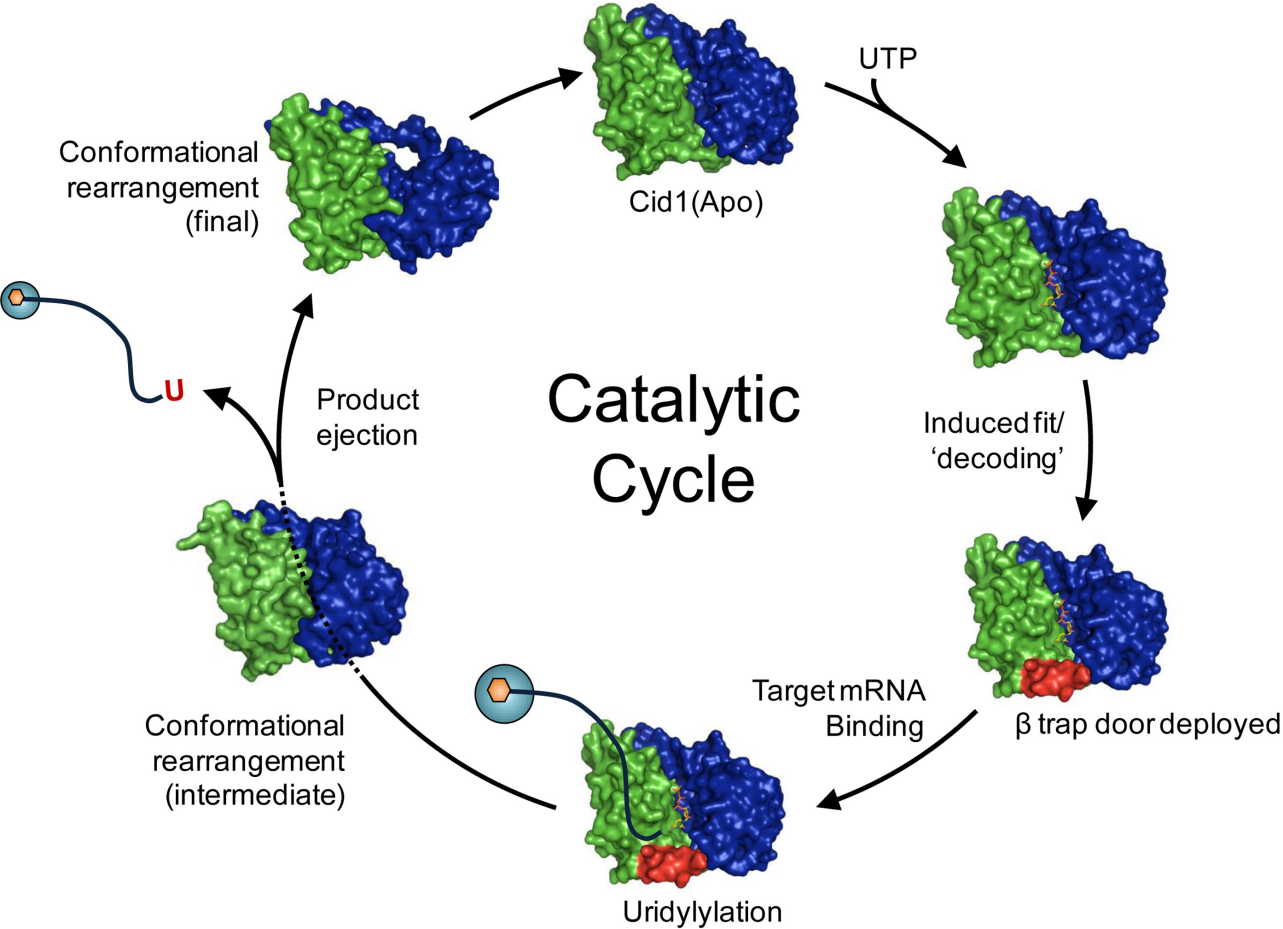
A proposed catalytic cycle of Cid1. The Apo enzyme binds UTP via an induced fit mechanism. The deployment of a structured element, the β trap door, promotes UTP selection. RNA is bound on the surface of the enzyme via basic patches. A single uridylyl ribonucleotide is transferred to the 3′ end of the RNA and pyrophosphate is released. The uridylated RNA product is then ejected from the active site via a structural re-arrangement of the N-terminal domain.

Bringing together the insights gained so far into the conformational dynamics of Cid1 we can propose a model for its binding, uridylylation and release of RNA substrates which explains its distributive function on substrates with no or only a few 3′ terminal uridyl residues (22,41) (Figure 5). Binding of UTP and magnesium involves an induced fit mechanism triggered by the recognition of the uracil moiety by H336 in the context of the NRM (21) and the metal *via* a triad of catalytic aspartates (13–15). Next, small domain motions between the NTD and CTD result in stabilization of the β-trap door, which assists in substrate containment ((21) and this work). The binding of mRNA on the surface of the enzyme provides the second substrate which enters the active site and is then uridylylated at its 3′ end. Following nucleotidyltransfer the NTD rotates, bringing this domain into close proximity with the NRM and therefore any bound substrate/product. The closure of the active site is focused around the NRM and, given what is known about the position of the RNA 3′ end (22) and the path of the RNA substrate (21), it is plausible that specific interactions between the NTD and the NRM along with steric hindrance could orchestrate the dissociation of the product from the active site. Interestingly, the remainder of the active site in the closed form is solvent accessible, as indicated by a small channel filled with water molecules. This potentially allows the by-product, pyrophosphate, to leave the active site in the opposite direction to the RNA product after catalysis.

A detailed analysis of the closed enzyme reveals that the NTD motion brings F88, which is in van der Waals distance of the sugar in a UTP-bound enzyme, into close proximity (∼3.7 Å) of Y212, to mediate ring stacking interactions with the pyrimidine ring of UTP (Figure 2B). It is clear from the observed water molecules within the closed active site that the benzyl side chain of phenylalanine acts to exclude the solvent, thus forming a hydrophobic constriction along with Y212 and therefore could play a role in ejecting the product. A structural comparison between this ‘intermediate’ closed form and product-bound Cid1 ((22); pdb 4nku) also reveals that upon closure the F88 side chain occupies the same position as the ribose sugar of the uridine of the ApU molecule. Additionally, the side chain of R111 forms a salt bridge with the carboxylic acid side chain of E333 when the enzyme is closed (Figure 2B). E333 has been shown to contact the 3′ nucleotide of the RNA substrate via a water molecule (22) and is clearly important for the catalytic activity of Cid1 (21). Furthermore, N164, which directly contacts the terminal nucleotide at the 3′ end of the RNA (22), contacts E333 when the enzyme is closed (Figure 2B). Thus, the formation of a salt bridge between R111 and E333 and the interaction between N164 and E333 would serve to enhance dissociation between the enzyme and the RNA 3′ end and thus allow the product to leave the active site after nucleotidyltransfer. Product ejection returns the enzyme to the Apo state, ready for a new cycle.

## CONCLUSIONS

The structures reported in this paper provide further evidence for a mechanism of UTP selection by Cid1 based on dynamic flipping of the side-chain of H336, a mechanism which may also be found in other related TUTs such as ZCCHC6 and ZCCHC11. Furthermore, our structures provide another ‘snapshot’ in a product ejection mechanism, this time a more intermediate stage which also suggests a molecular basis for product dissociation.

Cid1 shows distributive activity towards polyadenylated transcripts *in vivo* (12) as opposed to the apparently processive activity observed *in vitro* and found with oligo-uridylated substrates (11,21,22). This difference can be explained by the law of mass action in *in vitro* conditions (Figure 4). However, the rapid association/dissociation rates (21) along with a lower affinity for poly(A) RNA (22) may also contribute to this phenomenon. The product ejection mechanism, via structural re-arrangement of the NTD and intra-protein interactions, may promote dissociation of the uridylylated RNA and perhaps, in part, explains the high dissociation rate observed if considered as part of the catalytic cycle (Figure 5). The metazoan cytoplasmic uridylyltransferases for which Cid1 provides an excellent model (8,21,42), are also distributive enzymes (43–45). The fact that altering the flexibility of the Cid1 NTD by site-directed mutagenesis converts it to a mono-uridyltransferase *in vitro* suggests a mechanism whereby the alteration of flexibility of the equivalent domains in ZCCHC6 and ZCCHC11 might convert them to oligo-uridyltransferases. This effect could be achieved by association with another protein or proteins. The pattern of mono- versus oligo-uridylylation in metazoans is determined in this way by the binding of the developmental regulator Lin28A (8,43–45) and Trim25 (46).

## ACCESSION NUMBERS

Atomic coordinates and structure factors have been deposited in the Protein Data Bank with accession codes 4ud4 and 4ud5.

## SUPPLEMENTARY DATA

Supplementary Data are available at NAR Online.

SUPPLEMENTARY DATA
