# Supplementary material for: Structural plasticity of Cid1 provides a basis for its distributive RNA terminal uridylyl transferase activity
Source: Nucleic Acids Res. 2015 Feb 20;43(5):2968–79. doi: 10.1093/nar/gkv122 (PMC4357723; doi:10.1093/nar/gkv122)
Supplement: SUPPLEMENTARY DATA [file supp_43_5_2968__index.html]

Structural plasticity of Cid1 provides a basis for its distributive RNA terminal uridylyl transferase activity — SUPPLEMENTARY DATA 

# Structural plasticity of Cid1 provides a basis for its distributive RNA terminal uridylyl transferase activity

## SUPPLEMENTARY DATA

**Files in this Data Supplement:**

- Supplementary Data
- Supplementary Movie 1
