# Supplementary material for: Structural plasticity of Cid1 provides a basis for its distributive RNA terminal uridylyl transferase activity
Source: Nucleic Acids Res. 2015 Feb 20;43(5):2968–79. doi: 10.1093/nar/gkv122 (PMC4357723; doi:10.1093/nar/gkv122)
Supplement: SUPPLEMENTARY DATA [file supp_gkv122_nar-03604-h-2014-File007.pdf]

Supplementary Data

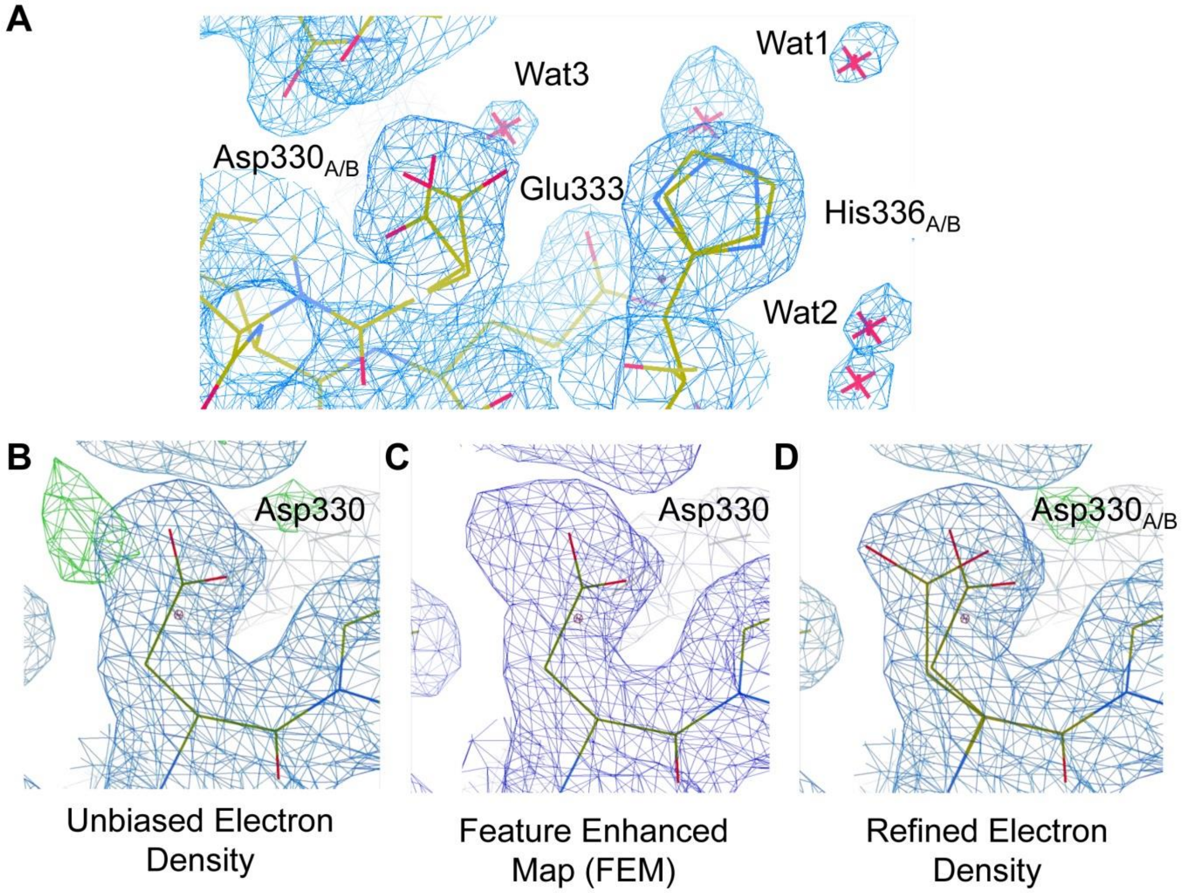

Supplementary Figure 1. Electron density around the nucleotide recognition motif. (A) Refined electron density ($2F_c$-F_o) of the Nucleotide Recognition Motif (NRM) showing a split conformation of the D330 and H336 side chain along with co-ordinated waters. Water molecules Wat1 and Wat2 are co-ordinated separately by a split H336 conformation. (B) Unbiased electron density (blue) showing a single conformation of D330 together with difference density (F_o-F_c; positive, green; negative, red) indicating the presence of another conformation. (C) Unbiased electron sharpened density using the Feature Enhanced Map (FEM; purple) routine implemented in Phenix (1). (D) Final refined density ($2F_o$-F_c) of D330. All maps were contoured to an r.m.s.d. of 1.00 and difference density contoured to an r.m.s.d. of 3.5.

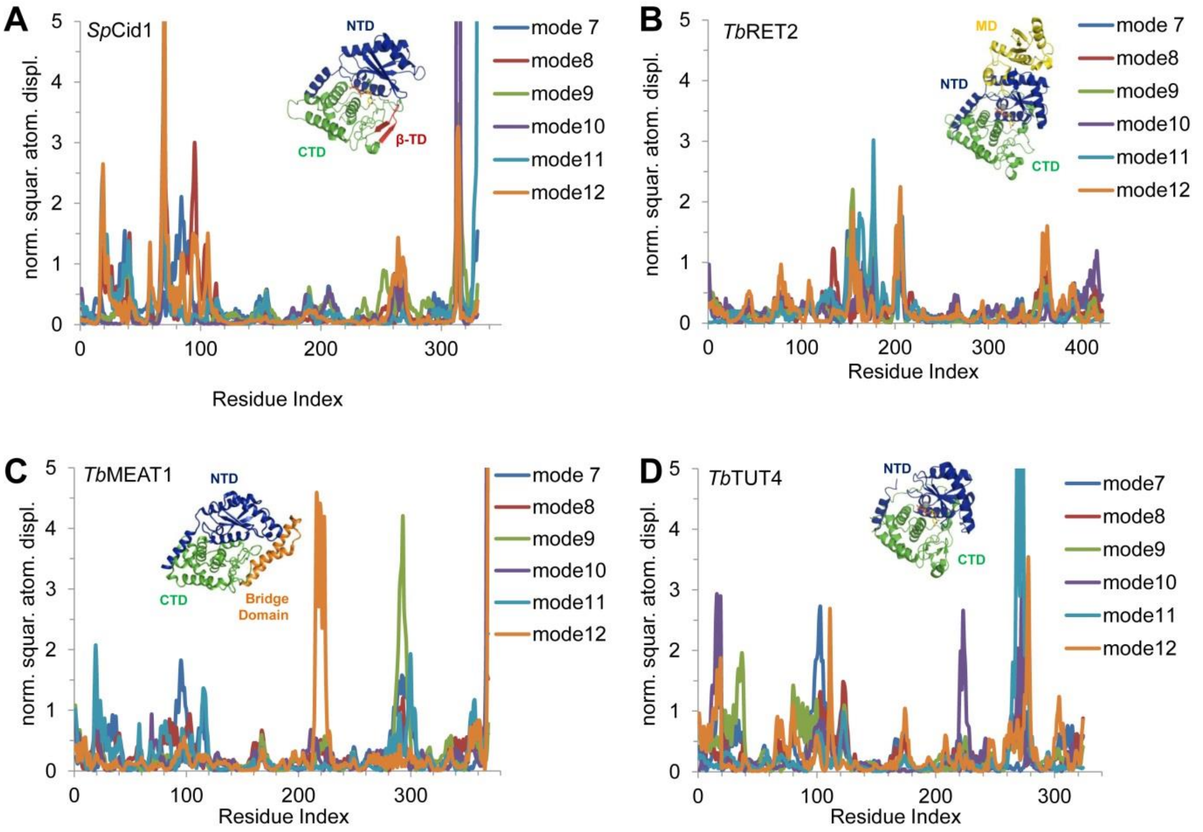

Supplementary Figure 2. Normal mode analysis atomic displacement (RMSD) of the six non-trivial modes for related Terminal Uridylyl Transferases (TUTs) structures calculated by WEBnm@ (2) for (**A**) Cid1 (pdb 4e80, chain C); (**B**) RNA editing TUTase2 (RET2) (pdb 2b4v, chain A); (**C**) Minor editosome associated TUTase1 (MEAT1) (pdb 3hj4, chain A); (**D**) Terminal Uridylyl Transferase 4 (TUT4) (pdb 2q0f, chain A).

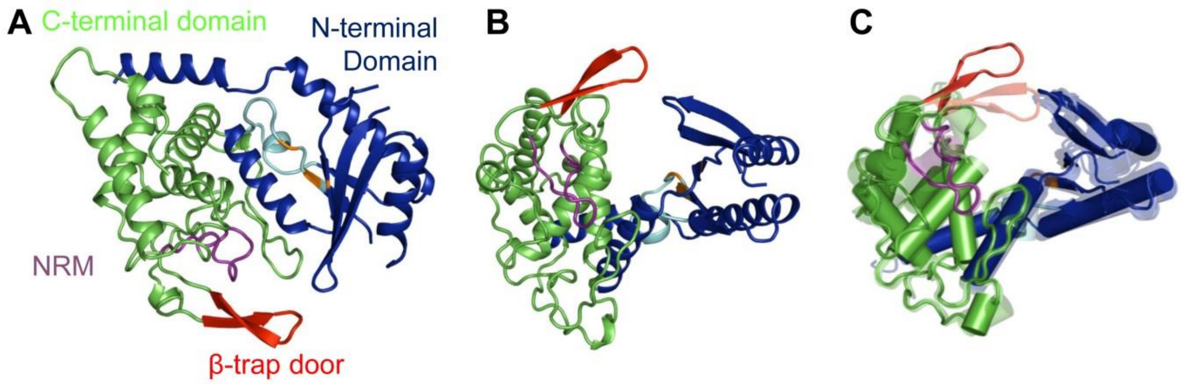

Supplementary Figure 3. A $P2_1$ Cid1 crystal structure with an ordered β-trap door. (**A**) Cid1 crystal structure coloured as in Figure 2A. (**B**) View of Cid1 rotated 90° from (A). (**C**) A superposition of all Cid1 crystal structures from the protein database (pdb) including those presented in this paper showing flexibility of the NTD and the difference between the two conformations of the β-trapdoor.

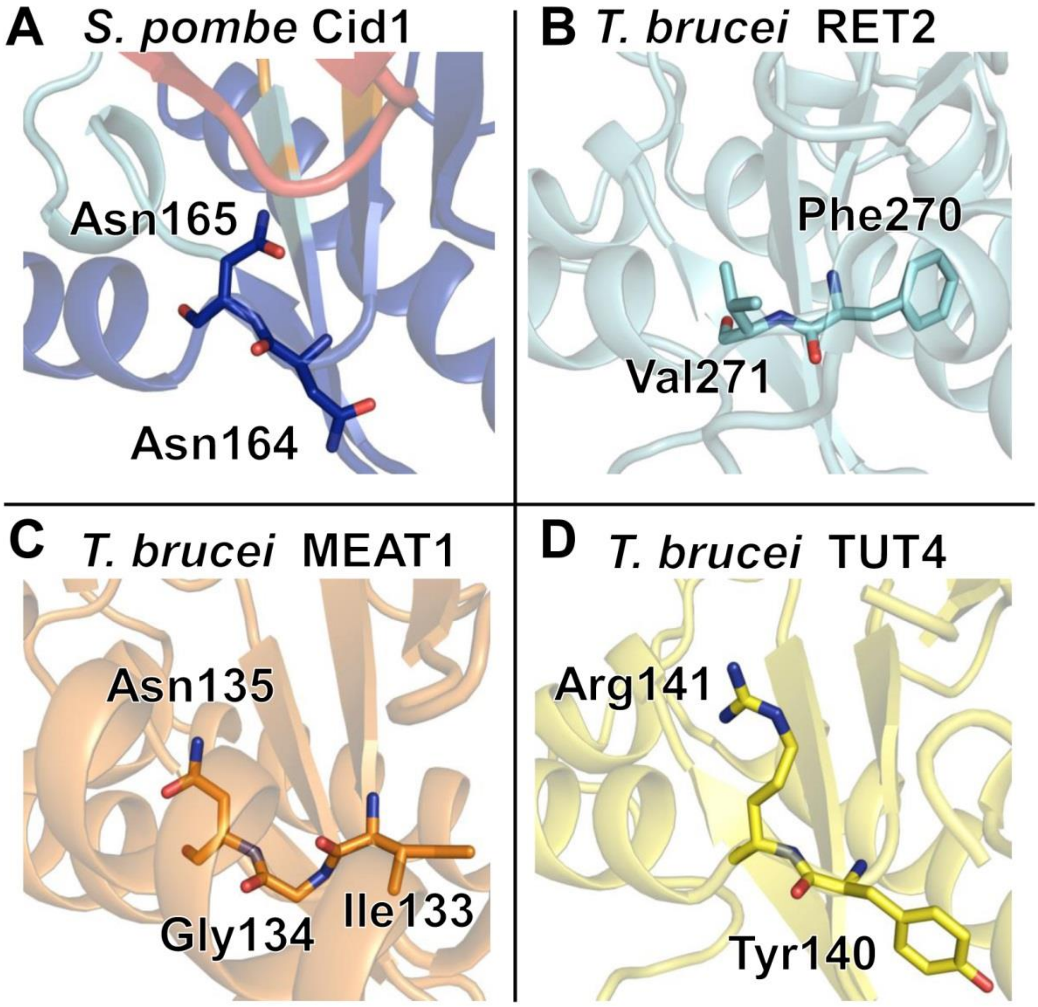

Supplementary Figure 4. Comparison of the hinge regions from other Terminal Uridylyl Transferases (TUTs). (**A**) TUT Cid1 from *Schizosaccharomyces pombe* (*S. pombe*) was structurally aligned to (**B**) RET2 (**C**) the mitochondrial minor editosome-associated TUT1 (MEAT1) and (**D**) TUT4 from *Trypanosoma brucei.* In Cid1 N164-N165 represents a pivot point for domain motion and they are shown with sidechains displayed for clarity. The equivalent residues in the other TUTs are also shown for comparison.

Supplementary Movie 1. The movie was rendered using Chimera (UCSF) (3). The NTD is coloured blue, the CTD green and the movie morphs between all conformations described by us to date with critical amino acid sidechains shown in detail. Note in particular the flipping of H336 (bottom left sidechain displayed) and the cis-trans isomerisation of the N164-N165 hinge region (top pair of sidechains displayed).

Supplementary references

1. Afonine, P.V., Grosse-Kunstleve, R.W., Echols, N., Headd, J.J., Moriarty, N.W., Mustyakimov, M., Terwilliger, T.C., Urzhumtsev, A., Zwart, P.H. and Adams, P.D. (2012) Towards automated crystallographic structure refinement with phenix.refine. *Acta crystallographica*, **68**, 352-367.
2. Hollup, S.M., Salensminde, G. and Reuter, N. (2005) WEBnm@: a web application for normal mode analyses of proteins. *BMC bioinformatics*, **6**, 52.
3. Pettersen, E.F., Goddard, T.D., Huang, C.C., Couch, G.S., Greenblatt, D.M., Meng, E.C. and Ferrin, T.E. (2004) UCSF Chimera--a visualization system for exploratory research and analysis. *J Comput Chem*, **25**, 1605-1612.
